# Reviewed article: Carvalho, SMO; Andrade, JX; Nascimento, FF; Rodrigues, MTP; Mascarenhas, MDM. Temporal trends in acquired syphilis detection, hospitalizations, and mortality: a time series analysis, Brazil, 2011-2023. Epidemol Serv Saude. 2026:35;e20251388

**DOI:** 10.1590/S2237-96222026v35e20251388.b

**Published:** 2026-11-02

**Authors:** Iara Beatriz Andrade de Sousa

**Affiliations:** 1 Empresa Brasileira de Serviços Hospitalares, Dourados, MS, Brazil Empresa Brasileira de Serviços Hospitalares Dourados MS Brazil

## Peer review B

### Questionnaire

Does the manuscript contain new and significant information to justify publication? Yes 

Does the Abstract (Summary) clearly and accurately describe the content of the article? Yes 

Is the problem significant and concisely stated? Yes 

Are the methods described comprehensively? No

Are the interpretations and conclusions justified by the results? Yes 

Is adequate reference made to other work in the field? Yes 

### Manuscript Structure:

Length of article is: Adequate

Number of tables is: Adequate

Number of figures is: Adequate

### Rating

Interest: Good

Quality: Good

Originality: Average

Overall: Good

Recommendation: Minor Revision 

### Comments

[1] Clareza da base populacional utilizada nas análises.

Os métodos estatísticos utilizados aparentam ser adequados ao delineamento do estudo. Entretanto, não ficou claro qual base populacional foi utilizada para o cálculo das taxas em cada ano analisado. Nas linhas 18-49 página 4, menciona-se a população brasileira estimada pelo IBGE para julho de 2024, embora o estudo não contemple esse período na análise. Considerando que o denominador populacional interfere diretamente nos resultados, a utilização de uma única estimativa populacional pode subestimar os valores dos anos iniciais da série histórica, especialmente devido ao crescimento populacional ao longo do tempo. Mesmo os autores descrevendo no parágrafo (linhas 20-29 pg 5) que utilizaram a população residente no mesmo local e período, não está claro quais estimativas foram efetivamente empregadas para cada ano e região geográfica. Sugere-se esclarecimentos quanto aos números populacionais utilizados para cada região geográfica e ano.

[2] Organização e objetividade dos resultados.

A seção de resultados necessita de revisão para garantir clareza, precisão e objetividade. Os achados devem ser apresentados de forma organizada e lógica evitando repetições excessivas entre texto e tabelas. Atualmente, grande parte das informações descritas no texto reproduz integralmente os dados da Tabela 1. Recomenda-se priorizar os principais achados no texto e utilizar as tabelas como suporte complementar. Sugere-se ainda revisar a padronização de pontuação, espaçamento e apresentação dos dados estatísticos (VPA e IC), garantindo conformidade com os padrões científicos do periódico.

[3] Adequação da linguagem científica.

Em alguns trechos da discussão foram utilizados termos como “expansão consistente” (linha 52) e “imperativo” (página 10, linha 8). Recomenda-se revisar o texto para reduzir o uso de expressões interpretativas ou excessivamente enfáticas que não estejam diretamente sustentadas pelas análises estatísticas realizadas.

